# Functional characterization of porcine *septin12* and its role in male reproduction

**DOI:** 10.5713/ab.250538

**Published:** 2026-04-02

**Authors:** Pei Wang, Xia Zhang, Hailong Huo, Luogang Li, Feidi Wen, Shuyan Wang, YongYun Zhang, Renaguli Turgong, Jinlong Huo

**Affiliations:** 1 College of Animal Science and Technology, Yunnan Agricultural University, Kunming, China; 2 Department of Biological and Food Engineering, Lyuliang University, Lvliang, China; 3 Yunnan Open University, Kunming, China; 4 Teaching Demonstration Center of the Basic Experiments of Agricultural Majors, Yunnan Agricultural University, Kunming, China

**Keywords:** Expression Profile, Male Reproduction, Pig, Protein Interaction, Septin12, Subcellular Localization

## Abstract

**Objective:**

The objective is to identify and functionally characterize the pig *septin12* gene, focusing on its expression pattern, subcellular localization, and interacting proteins, in order to explore its potential roles in male reproduction.

**Methods:**

Full-length *septin12* cDNA was obtained from the testis of the Banna mini-pig inbred line (BMI) using rapid amplification of cDNA ends. Quantitative real-time polymerase chain reaction and western blot were employed to assess tissue-specific expression in BMI pigs. Subcellular localization was determined by fluorescence microscopy following transfection of EGFP-tagged septin12. A yeast two-hybrid (Y2H) screen using an adult BMI testis cDNA library identified potential interacting proteins, which were then validated by Co-immunoprecipitation (Co-IP) and immunofluorescence (IF). Gene Ontology (GO) analysis was performed to ascertain the functional categories of the interactors.

**Results:**

The cloned *septin12* cDNA from the BMI testis was 1,289 base pairs (bp) in length. Septin12 mRNA and protein expression was predominantly observed in the BMI testis, suggesting its critical role in male reproductive function. Septin12 localized to both fibrous filamentous and punctate cytoplasmic structures. Thirteen interacting proteins were identified by Y2H, including ATAD5, ATP1B3, CENPL, ENO1, and septin5, with GO analysis indicating enrichment in mitotic nuclear division, cell growth, and G2/M phase transition. Furthermore, Co-IP and IF confirmed interactions between septin12 and electron transfer flavoprotein subunit alpha (ETFA) as well as lactate dehydrogenase B (LDHB), supporting the results of the Y2H screen.

**Conclusion:**

This study identified and characterized the *septin12* gene in BMI pig, highlighting its predominant expression in the testis and implicating its role in male reproductive function. Protein interaction analysis uncovered partners involved in cell division and metabolism, suggesting potential mechanisms by which *septin12* may influence spermatogenesis. Co-IP and IF further validated interactions with ETFA and LDHB. These findings offer a groundwork for future investigations on the role of *septin12* in male fertility.

## INTRODUCTION

Septin12, a member of the *septin* gene family, was first identified in *Saccharomyces cerevisiae* as a key component of the cytoskeleton [1]. In mice, septin12 is localized in the cytoplasm and nucleus of spermatocytes and round spermatids, as well as in the flagella of elongating spermatids [2]. *Septin12*^+/−^ chimeric mice exhibit significant spermatogenesis defects, including sperm immotility, tail curvature, round head, and acrosome abnormalities [3]. In humans, septin12 is found at the nuclear periphery of spermatid, the sperm neck, mitochondrion, and the spermatid ring [4]. It forms heteromeric septin complexes: with septin1/2/10/11 at the sperm neck and septin1/4/6/7 at the annulus, where it stabilizes the diffusion barrier between the midpiece and principal piece [5]. Crucially, septin12 interacts with cytoskeletal components such as α- and β-tubulins and partners with SUN5 to ensure proper head–tail junction in the spermatogenesis process [6].

cDNA microarray analyses of testicular tissues from fertile and infertile human males have highlighted *septin12* as a candidate gene potentially involved in male infertility [7]. Clinically, mutations in *septin12* have been associated with teratozoospermia and severe oligospermia. Notably, the c.474 G>A polymorphism in human *septin12* gene has been linked to sperm neck defects in teratozoospermic patients and acephalic sperm syndrome [8,9]. In addition, the human *septin12* gene c.673C>A (p.Gln225Lys) variant has been reported in a case of non-obstructive azoospermia (NOA), implying that this SNP may contribute to spermatogenic failure [10]. Recent research has further implicated *septin12* as a causative gene in acrosomal defects and has demonstrated its association with human acrosomal dysplasia [11]. Collectively, these findings from human and mouse studies underscore the critical role of *septin12* in spermatogenesis, sperm morphology, and capacitation.

Although extensive research has been conducted on *septin12* in humans [11,12] and mice [13,14], where it plays essential roles in spermatogenesis and male fertility, its biological role in pigs remains largely unknown. This knowledge gap is significant because pigs differ from rodents in reproductive physiology and also serve as an important biomedical model. The Banna mini-pig inbred line (BMI), with over 45 years of selective inbreeding and a high genetic homogeneity [15,16], provides an ideal system for studying gene function with minimal genetic noise. To clarify the role of *septin12* gene in pigs, we systematically characterized its expression profile and cellular localization in BMI boars. Additionally, we identified and validated its interacting proteins using yeast two-hybrid (Y2H) screening, co-immunoprecipitation (Co-IP) and immunofluorescence (IF). Our findings provide new evidence of *septin12*-associated molecular networks in pigs, expanding current understanding beyond mouse and human models.

## MATERIALS AND METHODS

### Animals and tissue collection

Three 10-month-old boars from the BMI were obtained from Yunnan Agricultural University. All pigs were reared under identical environmental and dietary conditions in an insulated, unheated shed with a half-slatted floor, located at the BMI pig farm of Yunnan Agricultural University. Following humane slaughter, 15 different tissues were collected from each boar, including heart, liver, spleen, lungs, kidneys, stomach, brain, muscle, duodenum, colon, seminal vesicles, prostate, bulbourethral glands, testes, and epididymides. All samples were immediately snap-frozen in liquid nitrogen and stored at −80°C until further use. The testicular tissues were fixed in 4% paraformaldehyde for 24 h, followed by dehydration in a graded series of ethanol and xylenes and then embedded in paraffin.

### Cloning of pig *septin12* gene

Total RNA was extracted from porcine tissues using the RNAiso kit (TaKaRa). First-strand cDNA synthesis was performed using the M-MLV reverse transcription kit (Invitrogen). To amplify a partial cDNA of pig *septin12*, a primer pair (Septin12-CDS-F/R) was designed based on cattle *septin12* gene (NM_001098143.2) and porcine expressed sequence tags (EST) (CX058541.1, BI360561.1, CV873999.1 and CX061899.1) retrieved from the GenBank database (http://www.ncbi.nlm.nih.gov). Polymerase chain reaction (PCR) amplification was carried out under the following conditions: initial denaturation at 98°C for 1.5 min, 35 cycles of denaturation at 94°C for 30 s, annealing at 55°C for 30 s, and extension at 72°C for 1 min, followed by a final extension step at 72°C for 5 min. The PCR product was sequenced via Sanger sequencing and used to design gene-specific primers (GSP) for subsequent experiments (Table 1).

For 5’ and 3’ rapid amplification of cDNA ends (RACE), 1 μg of total RNA extracted from adult pig testes was used as the template. GSP and nested gene-specific primers (NGSP) were designed against the SMART II oligonucleotide (Clontech) and nested universal primer (NUP) sequences based on the obtained *septin12* cDNA sequence. A touchdown PCR protocol was applied for initial amplification, and the resulting product was diluted 20-fold to serve as the template for the second round. The second-round products were purified, cloned into the pMD-18T vector (TaKaRa), and sequenced for further analysis. The *septin12* gene cDNA sequences were aligned using Lasergene (DNAStar) and its molecular weight (MW) and isoelectric point (pI) were calculated using the ProtParam tool (https://web.expasy.org/protparam). Subcellular localization of the pig septin12 protein was predicted using the PSORT online tool (https://www.genscript.com/psort.html).

### Phylogenetic analysis

Septin12 protein sequences from humans and other vertebrates were retrieved from GenBank and aligned using ClustalW. Detailed sequence information is listed in Supplement 1. A multi-species phylogenetic tree was constructed using the neighbor-joining (NJ) method with 10,000 bootstrap replicates in MEGA 11 and visualized with iTOL (https://itol.embl.de/).

### Quantitative real-time polymerase chain reaction

Tissue-specific expression of *septin12* was performed using SYBR Premix Ex Taq (TaKaRa). Glyceraldehyde-3-phosphate dehydrogenase (*GAPDH*) was used as the internal reference gene (Table 1) and relative expression levels were calculated using the 2^−ΔΔCt^ method [17]. According to *septin12* expression across reproductive tissues (testis, epididymis, seminal vesicle, prostate, and bulbourethral gland), the bulbourethral gland showed the lowest expression level. Therefore, the bulbourethral gland was selected as the calibrator tissue (normalized to 1.0). Data were presented as mean±standard deviation (SD) and analyzed using GraphPad Prism 5.0. For comparisons among more than two groups, one-way ANOVA was applied, and Tukey’s post hoc multiple comparison test was used to assess differences between individual groups. Statistical significance was set at p<0.05.

### Western blot

Tissues were lysed in RIPA buffer containing protease and phosphatase inhibitors (R0010; Solarbio). Protein concentration was determined using the BCA assay (Beyotime Biotechnology). Protein lysates were electrophoresed on 10% sodium dodecylsulfate polyacrylamide gels and transferred to polyvinylidene difluoride (PVDF) membranes (IPVH00010; Merck Millipore). Membranes were blocked with 5% non-fat dried milk and incubated at 4°C overnight with primary antibodies against septin12 (1:700; 13707-1-AP; Proteintech) and beta-tubulin (1:5,000, 66240-1-Ig; Proteintech). After washing, membranes were incubated with Multi-rAb HRP-Goat Anti-Mouse Recombinant Secondary Antibody (H+L) (1:5,000, RGAM001; Proteintech) and Multi-rAb HRP-Goat Anti-Rabbit Recombinant Secondary Antibody (H+L) (1:5,000, RGAR001; Proteintech). Subsequently, membranes were incubated with Super ECL Plus reagents (S6009S; UElandy) and bands were visualized using a western blot detection system (Bio-Rad Laboratories).

### Single-cell RNA sequencing analysis of *septin12* expression in adult boar testes

To investigate the cell type–specific expression pattern of *septin12*, we reanalyzed publicly available single-cell RNA sequencing (scRNA-seq) data from adult boar testes (NCBI BioProject ID: PRJNA1172176). Raw sequencing data were processed using the Seurat package. Low-quality cells were stringently filtered to ensure data reliability. After log-normalization and feature scaling, dimensionality reduction was performed by principal component analysis (PCA). Cell–cell neighborhood relationships were constructed using the FindNeighbors function, and cluster-specific marker genes were identified with FindAllMarkers. Cell clusters were subsequently annotated based on canonical germ cell marker genes. Finally, *septin12* expression across the annotated cell types was visualized and quantitatively compared using violin plots.

### Subcellular localization

A 1,092 open reading frame (ORF) of porcine *septin12* was amplified using primers Septin12-C-F/R (Table 1), electrophoresed on a 1.5% agarose gel, purified, and inserted into the *Xho* I/*Eco*R I sites of the pEGFP-C1 vector (Clontech), resulting in a fusion of EGFP to the 5’ end of septin12 (Supplement 2, designated pEGFP-septin12). The vector was verified by double digestion with *Xho* I and *Eco*R I, which yielded two fragments consistent with the expected sizes: an empty vector fragment of approximately 3.9 kb and a *septin12* insert of approximately 1.1 kb (Supplement 2). The sequence of the construct was further confirmed by sequencing. Swine testis (ST) cells were cultured in DMEM (Gibco) supplemented with 10% fetal bovine serum (FBS) (Gibco) and 2% penicillin/streptomycin at 37°C in a 5% CO_2_ atmosphere. Transfection was performed at 60%–70% confluency. After 48 h, cells were stained with MitoTracker Red CMXRos (Invitrogen) for mitochondria and Hoechst 33342 (Beyotime) for nuclei. Fluorescent signals were observed and imaged using an inverted fluorescence microscope (Zeiss).

### Construction of a testis cDNA library

The Y2H cDNA library of BMI testis tissue was constructed following the manufacturer’s instructions (ProQuest Two-Hybrid System Handbook). Briefly, total RNA was extracted from BMI testis using the RNeasy Mini Kit, and double-stranded cDNA was synthesized using the SMART technique. The porcine testis cDNA library for Y2H was generated through homologous recombination in yeast. To determine the recombination rate and inserted fragment sizes, 24 colonies were randomly selected from the cDNA library to amplify with primers pGADT7-F1/R1 (Table 1).

### Screening of yeast two-hybrid library

The bait plasmid (pGBKT7-septin12) was constructed using the pGBKT7 vector (Clontech) and amplified by PCR with a pair of specific primers containing the restriction sites *Eco*R I and *Sal* I (Table 1). Self-activation and functional verification of the bait pGBKT7-septin12 were conducted, and the Y2H library was screened using this bait. A fresh colony of the bait strain (Y2H Gold [pGBKT7-septin12]) was inoculated into SD/-Trp liquid medium and incubated at 30°C with shaking until the OD_600_ reached 0.8. The cells were then centrifuged and resuspended in SD/-Trp to achieve a density of >1×10^8^/mL, and then combined with the library strain (AD bacterial solution). After adding 2×YPDA liquid medium, the flask was incubated at 30°C for 20 h. The mating solution was examined under a microscope for trilobite-shaped conjugates. After confirmation mating, the cells were centrifuged and rinsed with YPDA. The mated culture was diluted 1,000-fold, and resuspension was plated on triple dropout medium (low stringency selection), incubating at 30°C for 3–5 days. Blue colonies were selected and replated on quadruple dropout plates (high stringency, SD/-Trp/-Leu/-Ade/-His/X-α-Gal/-AbA) containing X-α-Gal. Yeast plasmids were extracted using a yeast plasmid kit (DP112; TIANGEN) and target inserts were validated by sequencing with primers pGADT7-F2/R2 (Table 1). The pGADT7-F2 primer corresponds to the T7 forward primer, and pGADT7-R2 corresponds to the 3’ AD reverse primer. Furthermore, to validate the interaction between septin12 and cellular proteins, the bait and prey plasmids were co-transformed into the Y2H Gold yeast strain and incubated at 30°C for 3–5 days. Identified protein sequences were further analyzed using the PFAM (https://www.ebi.ac.uk/interpro/) and SMART (https://smart.embl.de) databases to assess their function domains. To better investigate the biological functions of septin12-interacting proteins, we conducted enrichment analyses of Gene Ontology (GO) terms using DAVID (https://davidbioinformatics.nih.gov/tools.jsp).

### Co-immunoprecipitation

To validate the interactions between septin12 and lactate dehydrogenase B (LDHB), as well as septin12 and electron transfer flavoprotein subunit alpha (ETFA), HEK293T cells were transfected with the HA-tagged septin12 plasmid (pGADT7-septin12), and harvested 48 h post-infection. Plasmid pGADT7-septin12 was constructed using the pGADT7 vector (Clontech) and amplified by PCR with a pair of specific primers containing the restriction sites *Nde* I and *Eco*R I (Table 1). The cells were washed with cold phosphate-buffered saline (PBS) and lysed with Western and IP cell lysis solution (P0013; Beyotime). After the whole cell harvest, immunoprecipitation was performed using anti-HA monoclonal antibody (AH158; Beyotime) in conjunction with protein A/G beads (sc-2003; Santa Cruz Biotechnology). The immunoprecipitated complex was washed with TBST buffer and then resuspended in SDS-PAGE protein loading buffer. The supernatant was subsequently subjected to Western blot analysis following standardized procedures using anti-LDHB (SAB4300603, 1:2,500; OriGene), anti-ETFA (C3956, 1:2,000; LSB), anti-β-actin (AF2811, 1:6,000; Beyotime), and HRP-conjugated secondary antibodies (bs-0295G-HRP, 1:5,000; Bioss). β-actin was used as the internal control. Protein bands were visualized using BeyoECL Plus Chemiluminescence Reagent (P0018S; Beyotime) and digitally captured through a chemiluminescence detection system (ChemiDoc MP Imaging System; Bio-Rad Laboratories).

### Immunofluorescence

Testis cross-sections (5 μm) from BMI boars were deparaffinized. The slides were treated with 0.5% Triton X-100 in PBS for 5 min, washed three times with PBS, incubated for 60 min with BSA solution. Anti-septin12 antibody (1:100, 13707-1-AP; Proteintech), anti-ETFA antibody (1:100, 12262-1-AP; Proteintech) and anti-LDHB antibody (1:100, 14824-1-AP; Proteintech) were diluted in the blocking solution and applied to the slides overnight at 4°C. The slides were washed three times with 0.03% TritonX-100 in TBS, treated with Cy3- and FITC-conjugated secondary antibody (GB21303, GB22303; Servicebio) for 2 h, and washed with 0.03% TritonX-100 in TBS. Coverslips were then mounted with Prolong-Gold reagent containing DAPI (Thermo Fisher Scientific) and visualized with fluorescence microscopy.

## RESULTS

### Isolation and characterization of porcine *septin12* gene

**Figure 1:**
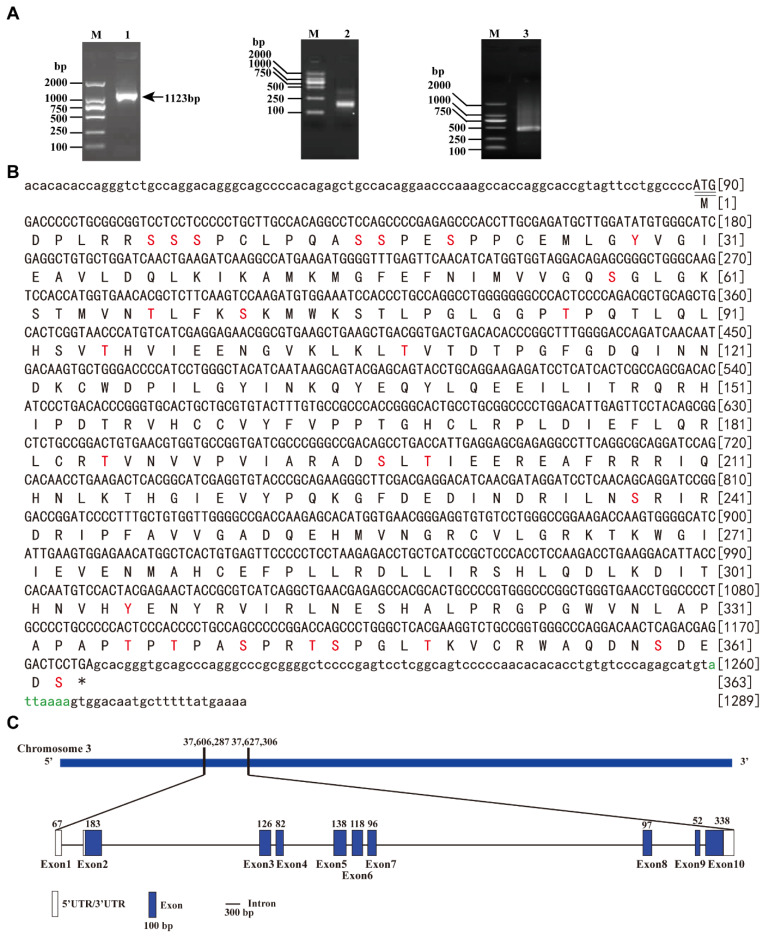
Isolation of the *septin12* gene from Banna mini-pig inbred line. (A) PCR amplification of the *septin12*. Lane M: DL2000 marker; lanes 1, 2, and 3 correspond to the RT-PCR product, second round 5’RACE product, and second round 3’RACE product, respectively. (B) The nucleotide and amino acid sequences of *septin12* gene. ATG is the start codon; * is the stop codon; Uppercase letters represent CDS, with the corresponding amino acid sequences shown below. Poly(A) signals (attaaaa) are highlighted in green, and predicted phosphorylation sites are marked in red. (C) Chromosome location and gene structure of *septin12*. Blue bars represent exons, and black lines indicate introns. PCR, polymerase chain reaction; RACE, rapid amplification of cDNA ends.

The *septin12* gene was amplified from BMI testis using RT-PCR, 5’RACE and 3’RACE techniques (Figure 1A). The full-length cDNA of *septin12* was 1,289 bp (GenBank No.: JX477173), comprising an 88 bp 5’ untranslated region (UTR), a 1,092 bp ORF, and a 118 bp 3’ UTR with a typical polyadenylation signal (attaaaa) at position 1,261, just 19 bp upstream from the poly (A) tail (Figure 1B). Structural analysis of the BMI septin12 protein (GenBank no. AFS88928.1) indicated a MW of 41.0 kD, and a molecular formula of C_1816_H_2890_N_522_O_529_S_19_. In total, 26 potential phosphorylation sites were identified, including 14 serine, 10 threonine, and 2 tyrosine residues (Figure 1B). The pI was 7.62. To elucidate the splicing of the pig *septin12* gene, the full-length cDNA sequence was aligned with the genomic sequence (*Sscrofa* 11.1). The *septin12* gene was located on the “+” strand of *Sus scrofa* chromosome 3, spanning 21,019 bp from 37,606,287 to 37,627,306, and contained 10 exons (1–10) and 9 introns (Figure 1C). The presence of 5’-GT and 3’-AG sequences at the end of each intron indicated that the *septin12* sequence adhered to the GT-AG boundary rule.

### Phylogenetic analysis

**Figure 2:**
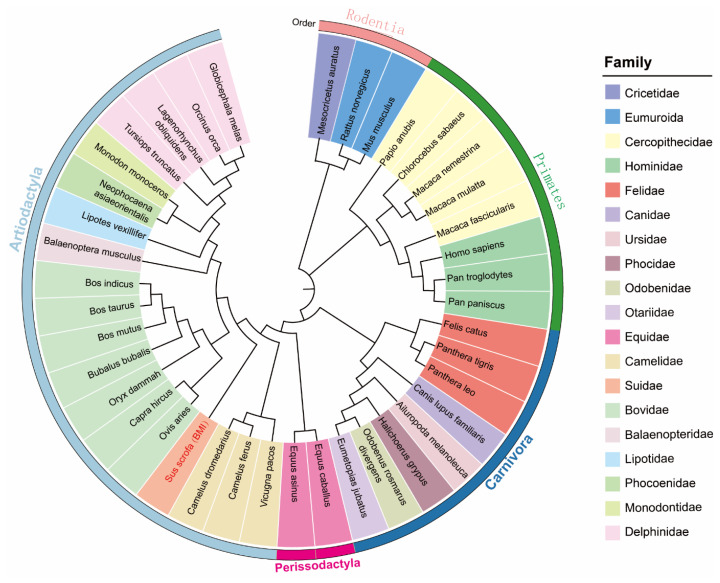
Phylogenetic tree of septin12 across 40 mammalian species constructed using the neighbor-joining method.

To assess the evolutionary relationship of septin12 between species, a phylogenetic analysis was conducted using the protein sequences from 40 species, including *Sus scrofa* (BMI) and 39 mammalian species obtained from the GenBank database. The NJ method was applied for clade support assessment. As shown in Figure 2, the sequences clustered into five main groups: *Rodentia*, *Primates*, *Carnivora*, *Perissodactyla*, and *Artiodactyla*. The BMI was positioned within the *Artiodactyla* cluster, alongside sequences from zebu, cattle, wild yak, water buffalo, scimitar oryx, goat, and sheep.

### Expression pattern of *septin12* gene

**Figure 3:**
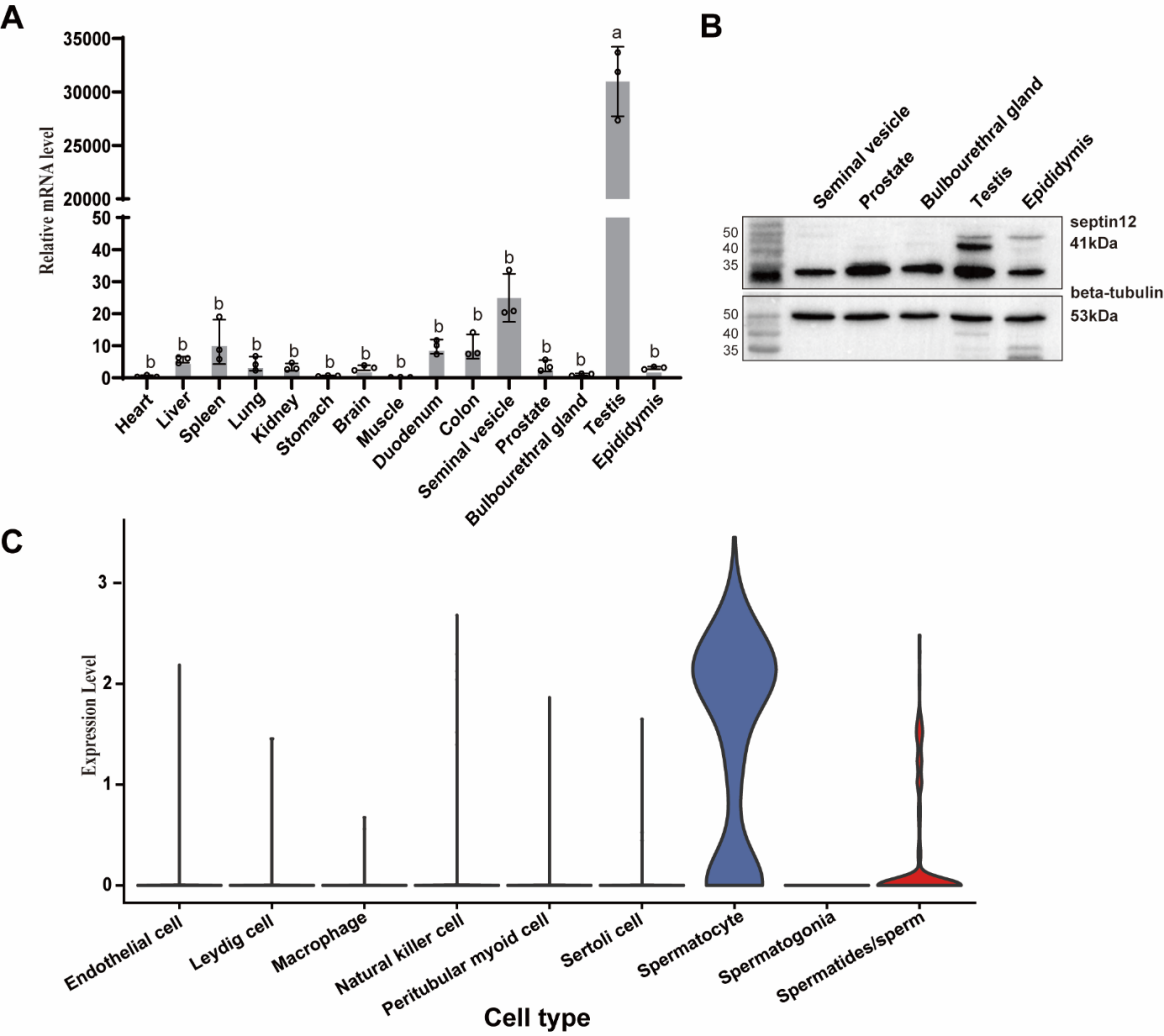
The expression profile of *septin12* gene in pig tissues. (A) qPCR result; The relative mRNA level in the bulbourethral gland was used as 1. (B) The Western blot result. Beta-tubulin was used as reference. (C) Expression of the *septin12* gene was assessed using single-cell RNA sequencing of adult boar testicular samples. ^a,b^ Different letters indicate significant differences (p<0.05). qPCR, quantitative real-time polymerase chain reaction.

The quantitative real-time PCR (qPCR) (Figure 3A) and western blot results (Figure 3B) revealed that septin12 was predominantly expressed in the testis of adult BMI pigs, with a prominent immunoreactive band detected at approximately 41 kDa, corresponding to the predicted MW of septin12. In addition, a lower-molecular-weight band (~32 kDa) was observed across multiple tissues (Figure 3B), which is likely attributable to nonspecific binding of the polyclonal septin12 antibody. To further verify the expression pattern of septin12 in pig testis, we analyzed the single-cell RNA sequencing data of adult boar testicular samples. Results (Figure 3C) showed *septin12* is mainly expressed in spermatocyte and spermatids/sperm.

### Subcellular localization of septin12 protein

**Figure 4:**
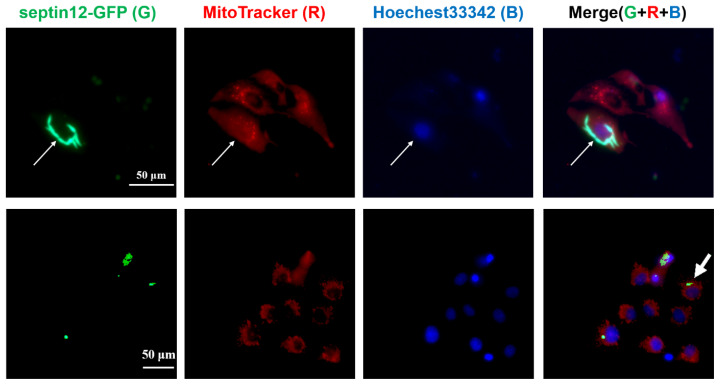
Subcellular localization of porcine septin12 protein. Swine testis (ST) cells were transfected with the pEGFP-septin12 vector, and the mitochondria and nuclei were stained with MitoTracker and Hoechst 33342, respectively. Septin12 displayed two distribution patterns: aggregated fibrous filaments (indicated by white thin arrow) and punctate formations (indicated by white thick arrow).

To investigate the subcellular distribution of the porcine septin12 protein, we expressed the pEGFP-septin12 vector in ST cells. Fluorescence microscopy results (Figure 4) demonstrated that the septin12 protein exhibited a distinctive speckled distribution around the nucleus, characterized by two predominant localization patterns: aggregated fibrous filaments and punctate formations. The PSORT prediction indicated that the pig septin12 protein is most likely localized to the mitochondria (43.5%) and the cytoplasm (21.7%), with lower probabilities assigned to other compartments. These predicted localization patterns were basically consistent with our fluorescence-based subcellular localization results, which also showed septin12 predominantly distributed in the cytoplasmic region. IF analysis showed endogenous pig septin12 protein is also predominantly localized in the cytoplasmic region of porcine testicular spermatocytes, and interstitial (Leydig) cells (Supplement 3).

### Yeast two-hybrid screening for septin12-interacting proteins

**Figure 5:**
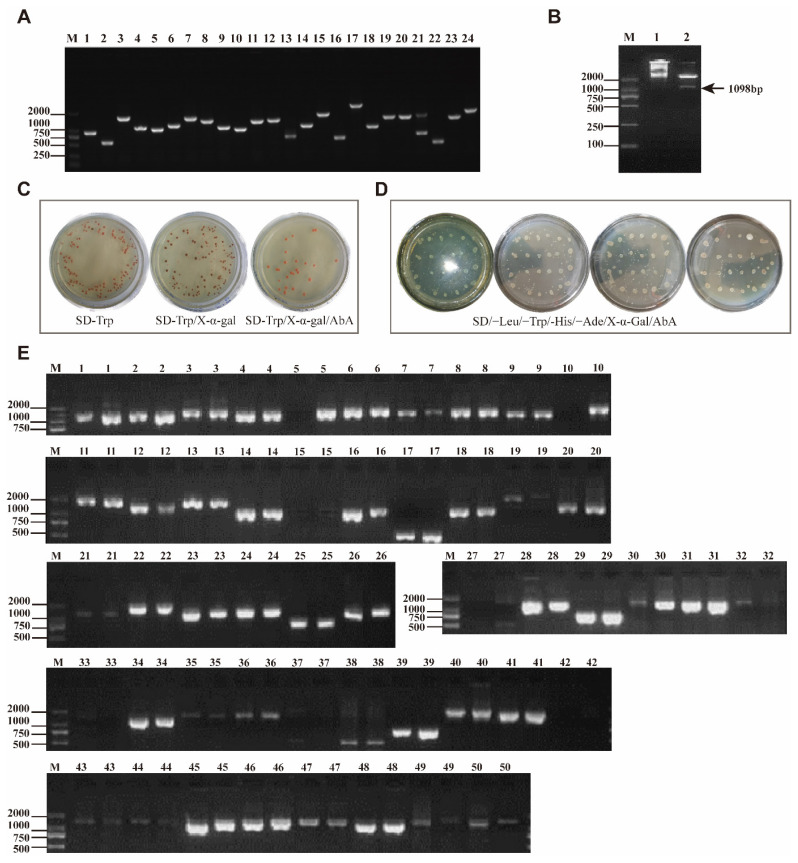
Identification of interactions between porcine septin12 and other proteins using the yeast two-hybrid system. (A) PCR identification of insert fragment recombination rate in Y2H library. (B) Double enzyme digestion confirmation of pGBKT7-septin12. (C) Verification of pGBKT7-septin12 autoactivation. (D) Yeast colonies that grow on the tetrad plates. (E) Positive colonies were identified by agarose gel electrophoresis (lanes 1–50). PCR, polymerase chain reaction; Y2H, yeast two-hybrid.

A cDNA library was constructed using RNAs isolated from adult BMI pig testis. Most of the cDNA fragments in the library were greater than 1.0 kb, with a size range of 0.5–2.5 kb (Figure 5A). The primary library titer was approximately 8.0×10^6^ colony-forming unit (CFU), ensuring sufficient coverage of testis-expressed genes. The ORF of *septin12* was subcloned into the pGBKT7 vector to generate the bait plasmid (Figure 5B). To confirm that the pGBKT7-septin12 bait itself did not autonomously activate reporter genes or exhibit toxicity, the bait strain was plated on SD/-Trp, SD/-Trp/X-α-gal, and SD/-Trp/X-α-gal/AbA media. No blue colony growth (Figure 5C) was observed on the selective media, indicating the bait was suitable for use in the subsequent screen. The initial screening identified approximately 50 positive colonies capable of growing on SD/−Leu/−Trp/-His/−Ade/X-α-Gal/AbA yeast medium (Figure 5D). The appearance of blue and white colonies confirmed interactions between the bait pGBKT7-septin12 and the prey pGADT7, simultaneously activating the *MEL1* and *AbAr* reporter genes. Each positive colony was isolated for PCR verification (Figure 5E) and sequencing using the primers pGADT7-F2 and pGADT7-R2 (Table 1). After alignment and removal of repetitive sequences, 18 unique cell proteins were identified as interactors with the BMI septin12 protein.

### Validation of septn12-interacting proteins

To validate the initial screening results, a secondary screening was performed using one-to-one interactions between the prey and the bait plasmid. The 18 pGADT7 prey clones identified in the first screen were individually transformed into yeast with the pGBKT7-septin12 bait plasmid and plated on nutrient-deficient QDO (SD/−Leu/−Trp/-His/−Ade) media. Positive colonies showed typical blue colonies, confirming the activation of the selection markers controlled by the *GAL4* promoter (Supplement 4). Subsequently, thirteen annotated cDNAs (ATAD5, ATP1B3, CENPL, E4F1, ETFA, ENO1, GMCL1, LDHB, MRPS9, septin5, TOM1L1, WFS1, and ZNF251) were identified as positive interactors (Supplement 4), suggesting their interaction with pig septin12.

**Figure 6:**
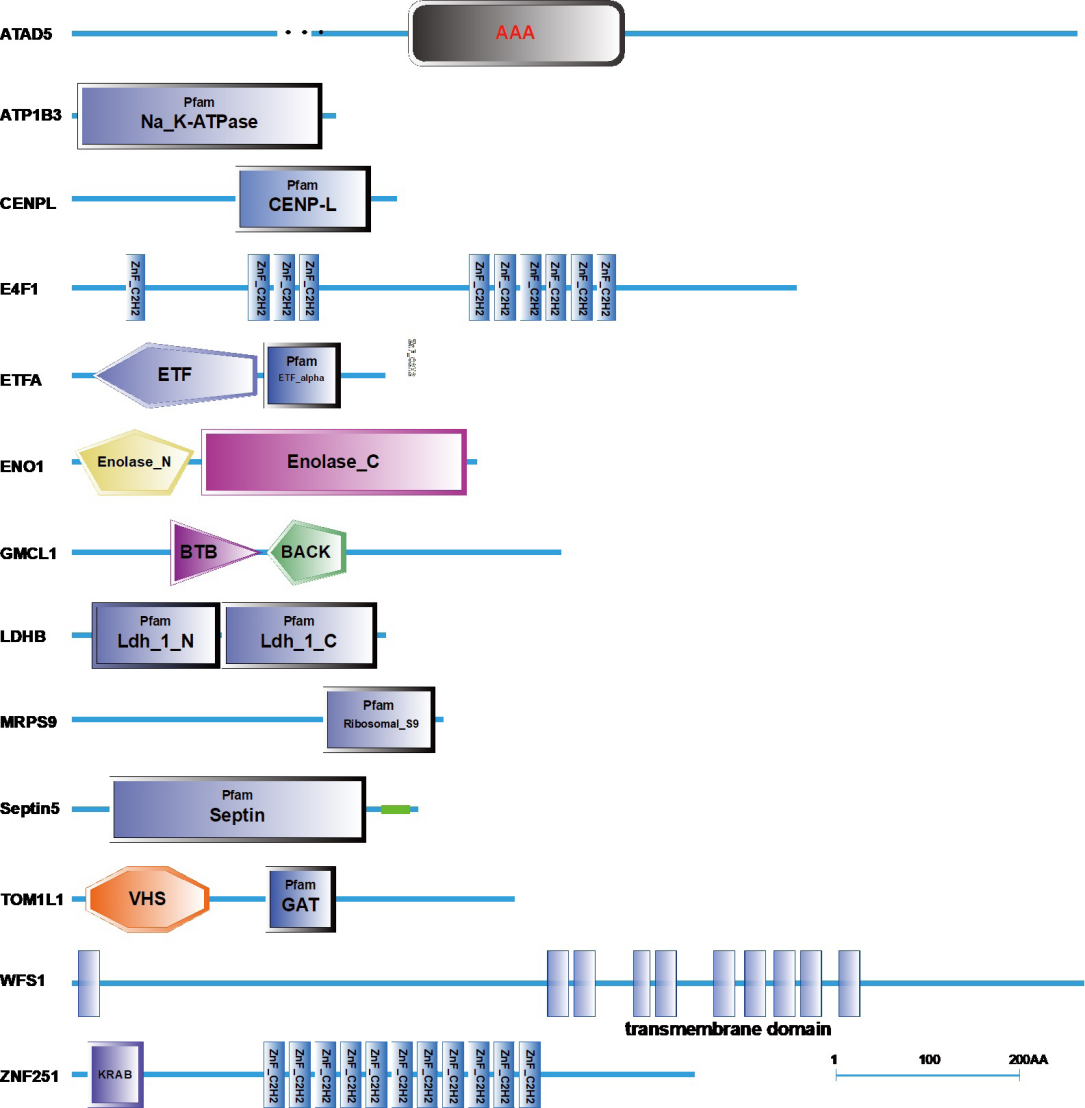
Schematic presentation of the domain structures of the 13 proteins that interact with porcine septin12. The identified domains: AAA, ATPases associated with diverse cellular activities; Na_K-ATPase, sodium/potassium-transporting ATPase subunit beta; CENP-L, Centromere protein L; ZnF_C2H2, C2H2-type (classical) zinc fingers; ETF, Electron transfer flavoprotein domain; ETF_alpha, Electron transfer flavoprotein FAD-binding domain; Enolase_N, Enolase N-terminal domain; Enolase_C, Enolase C-terminal TIM barrel domain; BTB, Broad-Complex, Tramtrack and Bric a brac; BACK, BTB and C-terminal Kelch; Ldh_1_N, N-terminal of L-lactate dehydrogenases; Ldh_1_C, C-terminal of L-lactate dehydrogenases, Ribosomal_S9, ribosomal protein S9; VHS, domain present in VPS-27, Hrs and STAM; GAT, GGA protein binding domain for ARF family members; KRAB, Krueppel associated box.

**Figure 7:**
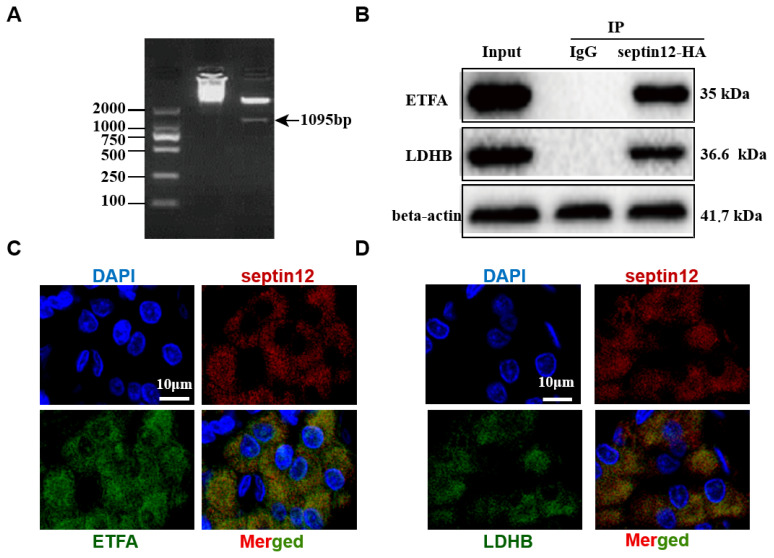
Validation of septn12 interacting proteins. (A) Confirmation of pGADT7-septin12 by double enzyme digestion. (B) Co-IP assay for septin12 interactions. HEK-293T cells were transfected with plasmids expressing HA-septin12. Cell lysates were immunoprecipitated using IgG and anti-HA antibodies, followed by immunoblotting with anti-ETFA and anti-LDHB antibodies. The first lane represents the input group, the second lane is the IgG control group, and the third lane is the experimental group. (C) Immunofluorescence images of septin12 (red) and ETFA (green) in porcine testis sections. (D) Immunofluorescence images of septin12 (red) and LDHB (green) in porcine testis sections. ETFA, electron transfer flavoprotein subunit alpha; LDHB, lactate dehydrogenase B; Co-IP, co-immunoprecipitation.

Protein domain architectures provide valuable information to determine protein functions. We further analyzed the domains of the 13 interacting proteins using the SMART and Pfam databases. A total of 18 types of domains were found in these proteins, including AAA, Na_K-ATPase, CENP-L, ZnF_C2H2, ETF, ETF_alpha, Enolase_N, Enolase_C, BTB, BACK, Ldh_1_N, Ldh_1_C, Ribosomal_S9, Ffam septin, VHS, GAT, transmembrane domain, and KRAB domains (Figure 6). GO analysis of these 13 proteins revealed their involvement in processes such as mitotic nuclear division, cell growth, cell cycle G2/M phase transition, mitotic recombination, amino acid catabolism, lactate metabolism, cytokinesis, protein stabilization, and germ cell development (Supplement 5). GO cellular component annotation (Supplement 5) showed five proteins (ETFA, LDHB, septin5, TOM1L1, and WFS1) shared cytoplasmic localization with septin12. Furthermore, GO biological process analysis (Supplement 5) revealed that ETFA and LDHB are involved in mitochondrial energy metabolism and glycolytic metabolism, respectively, both of which play critical roles in spermatogenesis and sperm motility. Given their functional relevance, ETFA and LDHB were selected for Co-IP and IF validation. A pGADT7-septin12 plasmid was constructed (Figure 7A) and Co-IP assays were performed in HEK293T cells. The results demonstrated that septin12 co-immunoprecipitated with both ETFA and LDHB, confirming their physical interaction (Figure 7B). In parallel, IF co-localization results (Figures 7C, 7D) showed similar subcellular localization patterns for these proteins, further supporting their potential interaction within shared cytoplasmic compartments.

## DISCUSSION

The *septin12* gene encodes a testis-enriched cytoskeletal GTPase with filament-forming capacity, playing an indispensable role in spermiogenesis and sperm structural integrity [2,3]. Currently, disruptions in the function of the *septin12* gene have been confirmed to cause male infertility in humans and mice [3,9,18]. Nevertheless, there have been no investigations to the pig *septin12* gene. Given the physiological and genetic similarities between pigs and humans, pigs provide an ideal model for studying human reproductive diseases [19]. In our research, we focused on molecular features, expression patterns, subcellular localization, and identification of septin12-interacting proteins in BMI pigs.

RACE is an effective method to amplify full-length mRNA by targeting specific sequences at the 3' or 5' ends of the transcript. In this study, we employed RACE to obtain the full-length cDNA sequence of BMI *septin12*. The resulting sequence revealed that the pig *septin12* had a 1,289 bp full-length mRNA encoding a protein of 363 amino acids. We identified ten exons and nine introns in the porcine *septin12* gene, similar to the human *septin12* transcript (*septin12*_201, ENST00000268231.13). Additionally, the multiple sequence alignment analysis revealed the high evolutionary conservation of septin12 among large mammals. Phylogenetic analysis grouped five main groups: *Rodentia*, *Primates*, *Carnivora, Perissodactyla*, and *Artiodactyla*. In the *Artiodactyla* cluster, pig, zebu, cattle, wild yak, water buffalo, scimitar oryx, goat, and sheep formed a distinct clade, consistent with the zoological classification. The high conservation observed across these species suggests that septin12 protein is functionally important and has evolved under strong evolutionary constraint.

Analyzing tissue-specific gene expression improves the understanding of its functional roles. Thus, we detected *septin12* expression across 15 tissues from BMI, revealing the highest expression levels in the testis, which is consistent with the results in humans and mice [3,4,20]. Specially, *septin12* mRNA was detected at low levels in the BMI seminal vesicle, whereas its protein (~41 kDa) was not observed in this tissue by western blot analysis. Notably, although an additional lower-molecular-weight band (~32 kDa) was observed in western blot analyses across multiple tissues, this signal lacked testis-enriched expression and is therefore likely attributable to nonspecific binding of the polyclonal septin12 antibody rather than a functional septin12 isoform. Together, these findings indicate that *septin12* expression in accessory reproductive glands may be subject to post-transcriptional regulation or represent a species-specific regulatory feature that merits further investigation. Consistent with this, pig scRNA-seq data show that *septin12* is predominantly expressed in spermatocytes and spermatids/sperm. Sperm hyperactive motility has been reported to influence litter size in pigs. Testicular RNA-seq analysis [21] further revealed that *septin12* is differentially expressed (FDR<0.05) between Landrace boars with high and low sperm hyperactive motility, providing preliminary transcriptomic evidence that *septin12* may play a role in porcine sperm function.

Determining the subcellular localization of a protein is crucial for investigating its interactions, functions, and potential roles across different cells. In this study, we constructed a vector pEGFP-septin12 to examine the subcellular localization of the septin12 protein in ST cells. We observed two typical distribution patterns: aggregated fibrous filaments and punctate patterns, potentially reflecting its diverse biological functions, which partially resemble the GFP-tagged septin12 pattern in HEK293T cells [6]. However, in Hela cells, septin12 was present in filamentous structures in the interphase cytoplasm, transferred to the central spindle and midbody during anaphase and cytokinesis, respectively [22]. In the Chinese Hamster Ovary (CHO) cell line, septin12 exhibited a septin-like expression pattern within the cellular context, localized to actin-based structures in both interphase and dividing cells [20]. These divergent patterns may indicate that the functional repertoire of septin12 is highly context dependent. In proliferating somatic cells such as HeLa and CHO, ectopically expressed septin12 likely contributes to cytokinetic fidelity and cortical rigidity, functions that are presumably shared among septin family members during cell division and morphogenesis [23]. Interestingly, although ST cells lack haploid spermatid structures, the filamentous/punctate pattern observed in these cells resembles the organizational pattern adopted by septin12 during spermiogenesis. One possibility is that certain germ cell-associated assembly behaviors of septin12 could be partially recapitulated even in testis-derived somatic environments. Notably, septin12 is predominantly expressed in the male germline rather than in somatic tissues, where it does not participate in cytokinesis but instead helps scaffold the sperm annulus and stabilize the head–tail junction, requiring precise spatial and temporal assembly distinct from somatic septins. However, these interpretations remain speculative and require further investigation.

Y2H screening offers molecular resources for analyzing protein-protein interactions based on known proteins. In this study, we constructed a Y2H library using RNA from the testis of adult BMI boar and assessed its quality by titre, recombination rate, and insert size. Our library storage capacity exceeded 8.0×10^6^ CFU, with a recombination rate of 100%, confirming the high quality of the library. Further, protein-protein interaction analysis using the pGBKT7-septin12 vector yielded 50 positive clones, and preliminary identified 18 interacting proteins by sequencing. We verified the interactions through one-to-one interaction between the prey and bait plasmids, finally confirming 13 proteins that interact with septin12. They were ATAD5, ATP1B3, CENPL, E4F1, ETFA, ENO1, GMCL1, LDHB, MRPS9, septin5, TOM1L1, WFS1, and ZNF251. *ATAD5* plays a crucial role in preserving genomic integrity in all organisms, including humans [24]. It is involved in the positive regulation of cell cycle G2/M phase transition, DNA clamp unloading, and positive control of DNA replication [25]. ATP1B3 is the β3 subunit of Na+/K+-ATPase, which is responsible for establishing and maintaining the electrochemical gradients of Na and K ions across the plasma membrane [26]. The centromere protein CENPL is part of the nucleosome-associated CENPA-NAC complex [27], which is essential for proper kinetochore function and mitotic progression [28]. E4F1 and ZNF251 both possess the classical zinc finger domain Znf_C2H2, which often acts as structural motifs that bind DNA or proteins, and therefore are crucial for cellular functions such as development, differentiation, and oncosuppression [29]. *ETFA* and *MRPS9* are involved in mitochondrial function and metabolism. ETFA participates in catalyzing the initial step of mitochondrial fatty acid beta-oxidation, defects in ETFA have been linked to type II glutaricaciduria [30]. Mitochondrial ribosomes, which participate in protein synthesis in mammals’ mitochondrion, are made up of a large 39S subunit and a tiny 28S subunit. *MRPS9* encodes a protein of the mitochondrial 28S subunit that is required for controlling mitochondrial activity and preserving structural integrity [31]. *ENO1* and *LDHB* are both associated with the glycolytic process. ENO1 is a key glycolytic enzyme that catalyzes the reversible conversion between 2-phosphoglycerate (2-PG) and phosphoenolpyruvate (PEP). Reactive oxygen species (ROS) stress has a profound impact on boar sperm motility and overall semen quality [32]. LDHB, the B subunit of lactate dehydrogenase (LDH), is a key glycolytic enzyme that catalyzes the interconversion of pyruvate and lactate [33]. By regulating cellular redox balance through NAD^+^/NADH turnover, LDHB plays an important role in maintaining intracellular ROS homeostasis. GMCL1 encodes a nuclear envelope protein, which is expressed at high levels in the testis. Loss of GMCL1 function can lead to markedly impaired fertility in mice, demonstrating a role for this gene in male germ cell development [34]. Septin5 and 12 were both members of the septin family formerly called cell division control related protein. Disruption of septin function disturbs cytokinesis and results in large multinucleate or polyploid cells [35]. Notably, CpG-binding protein 1 (CFP1) deficiency in mouse spermatocytes induces hypermethylation at the promoter regions of *septin* genes [36], suggesting that septins may serve as downstream targets of CFP1-mediated epigenetic regulation during spermatogenesis. TOM1L1 is a signaling adaptor protein that contains VHS and GAT domains. Protein transport between the trans-Golgi network and endosomes is facilitated by these regions [37]. The Wolframin protein, a transmembrane glycoprotein with nine transmembrane structural domains that are found mainly in the endoplasmic reticulum (ER) membrane, is encoded by the *WFS1* gene [38]. It plays an important role in the regulation of ER homeostasis. Variations in *WFS1* are responsible for Wolfram syndrome [39]. Although the GO analysis showed that the 13 septin12-interacting proteins are primarily associated with processes such as mitotic nuclear division, cell growth, cell cycle G2/M phase transition, mitotic recombination, amino acid catabolism, lactate metabolism, cytokinesis, protein stabilization, and germ cell development, the specific roles of most of these proteins in spermatogenesis remain undetermined. It will be interesting to investigate their relationships with septin12 during male pig reproduction in the future.

## CONCLUSION

In conclusion, this study provides the first comprehensive analysis of the molecular characterization, expression patterns, and subcellular location of the *septin12* gene in pigs. The full-length cDNA of BMI *septin12* spanned 1,289 bp, encoding a protein of 363 amino acids, showing high homology and evolutionary conservation with other mammals. Expression was predominantly detected in the testis. Subcellular localization exhibited aggregated fibrous filaments and punctate cytoplasmic patterns, suggesting roles in cell division, shape maintenance, and signal transduction processes. Thirteen interaction proteins were identified via Y2H assay, with GO analysis indicating functions related to mitotic division, cell growth, and G2/M phase transition. Interaction with ETFA/LDHB was further validated by Co-IP and IF. These findings provide a strong foundation for further exploration of the functional and regulatory mechanisms of *septin12* in pig reproduction. However, more thorough cellular and organismal studies are necessary to elucidate its precise molecular mechanisms.

## Figures and Tables

**Table 1:** All primers used in this study

Name	Primer sequence (5’–3')	Product size (bp)
Primer for CDS		
Septin12-CDS-F	AAAGCCACCAGGCACCGTAG	1,123
Septin12-CDS-R	TCAGGAGTCCTCGTCTGAGTT	
Primers for 5’RACE		
Septin12-GSP-R1	CGATGCCCACATATCCAAGCATCTC	
Septin12-NGSP-R2	GAACTACGGTGCCTGGTGGCT	
Primers for 3’RACE		
Septin12-GSP-F1	TGTCCTGGGCCGGAAGACCAAGTG	
Septin12-NGSP-F2	AAGTGGAGAACATGGCTCACTGTG	
Primers for qPCR		
Septin12-qPCR-F	CGGGACCGGATCCCCTTT	120
Septin12-qPCR-R	ACAGTGAGCCATGTTCTCCACTT	
GAPDH-qPCR-F	CCTTCATTGACCTCCACTACATGGT	183
GAPDH-qPCR-R	CCACAACATACGTAGCACCAGCATC	
Primers for subcellular location		
Septin12-C-F	CTCGAGCTATGGACCCCCTGCGGCGG (*Xho* I)	1,106
Septin12-C-R	GAATTCTCAGACGAGGACTCCTGA (*EcoR* I)	
Primers for yeast two-hybrid		
pGBKT7-septin12-F	GAATTCATGGACCCCCTGCGGCGGT (*EcoR* I)	1,104
pGBKT7-septin12-R	GTCGACTCAGGAGTCCTCGTCTGAGTT (*Sal* I)	
pGADT7-septin12-F	CATATGGACCCCCTGCGGCGGT (*Nde* I)	1,101
pGADT7-septin12-R	GAATTCTCAGGAGTCCTCGTCTGAGTT (*EcoR* I)	
pGADT7-F1	TAATACGACTCACTATAGGGCGAGCGCCGCCATG	
pGADT7-R1	GTGAACTTGCGGGGTTTTTCAGTATCTACGATT	
pGADT7-F2	TAATACGACTCACTATAGGGC	
pGADT7-R2	AGATGGTGCACGATGCACAG	

RACE, rapid amplification of cDNA ends; GSP, gene-specific primers; NGSP, nested gene-specific primers; qPCR, quantitative real-time polymerase chain reaction.

## Data Availability

Upon reasonable request, the datasets of this study can be available from the corresponding author.

## SUPPLEMENTARY MATERIAL

**Supplement 1**. Sequence information of septin12s in *Artiodactyla* and other vertebrates.

**Supplement 2**. Construction of co-expression vector pEGFP-septin12.

**Supplement 3**. Immunofluorescence staining of septin12 protein in pig testis sections.

**Supplement 4**. Result of point-to-point verification of the 13 prey plasmids and the bait plasmid pGBKT7-septin12.

**Supplement 5**. Gene ontology analysis on proteins interacting with septin12.

## References

[1] Mostowy S Cossart P Septins: the fourth component of the cytoskeleton Nat Rev Mol Cell Biol 2012 13 183 94 10.1038/nrm3284 22314400

[2] Kuo YC Shen YR Chen HI SEPT12 orchestrates the formation of mammalian sperm annulus by organizing core octameric complexes with other SEPT proteins J Cell Sci 2015 128 923 34 10.1242/jcs.158998 25588830

[3] Lin YH Lin YM Wang YY The expression level of septin12 is critical for spermiogenesis Am J Pathol 2009 174 1857 68 10.2353/ajpath.2009.080955 19359518 PMC2671274

[4] Yeh CH Kuo PL Wang YY SEPT12/SPAG4/LAMINB1 complexes are required for maintaining the integrity of the nuclear envelope in postmeiotic male germ cells PLOS ONE 2015 10 e0120722 10.1371/journal.pone.0120722 25775403 PMC4361620

[5] Shen YR Wang HY Kuo YC SEPT12 phosphorylation results in loss of the septin ring/sperm annulus, defective sperm motility and poor male fertility PLOS Genet 2017 13 e1006631 10.1371/journal.pgen.1006631 28346465 PMC5386304

[6] Zhang Y Liu G Huang L SUN5 interacts with nuclear membrane LaminB1 and cytoskeletal GTPase Septin12 mediating the sperm head-and-tail junction Mol Hum Reprod 2024 30 gaae022 10.1093/molehr/gaae022 38870534

[7] Lin YH Lin YM Teng YN Hsieh TYT Lin YS Kuo PL Identification of ten novel genes involved in human spermatogenesis by microarray analysis of testicular tissue Fertil Steril 2006 86 1650 8 10.1016/j.fertnstert.2006.04.039 17074343

[8] Özkara G Ersoy Tunali N SEPTIN12 c.474 G>a polymorphism as a risk factor in teratozoospermic patients Mol Biol Rep 2021 48 4073 81 10.1007/s11033-021-06417-7 34057684

[9] Dortaj S Gilani MAS Sabbaghian M Genetic investigations of SEPTIN12 gene in infertile men with acephalic sperm syndrome Gene Rep 2025 38 102130 10.1016/j.genrep.2025.102130

[10] Geng D Yang X Zhang H Association of single nucleotide polymorphism c.673C>A/p.Gln225Lys in SEPT12 gene with spermatogenesis failure in male idiopathic infertility in Northeast China J Int Med Res 2019 47 992 8 10.1177/0300060518811770 30488758 PMC6381467

[11] Li Y Wang Y Wen Y Whole-exome sequencing of a cohort of infertile men reveals novel causative genes in teratozoospermia that are chiefly related to sperm head defects Hum Reprod 2021 37 152 77 10.1093/humrep/deab229 34791246

[12] Miyakawa H Miyamoto T Koh E Single-nucleotide polymorphisms in the SEPTIN12 gene may be a genetic risk factor for Japanese patients with Sertoli cell–only syndrome J Androl 2012 33 483 7 10.2164/jandrol.110.012146 21636737

[13] Chen H Li P Du X Homozygous loss of Septin12, but not its haploinsufficiency, leads to male infertility and fertilization failure Front Cell Dev Biol 2022 10 850052 10.3389/fcell.2022.850052 35547809 PMC9082362

[14] Shen YR Wang HY Tsai YC The SEPT12 complex is required for the establishment of a functional sperm head–tail junction Mol Hum Reprod 2020 26 402 12 10.1093/molehr/gaaa031 32392324

[15] Huo JL Zhang LQ Zhang X Genome-wide single nucleotide polymorphism array and whole-genome sequencing reveal the inbreeding progression of Banna minipig inbred line Anim Genet 2022 53 146 51 10.1111/age.13149 34658041

[16] Chen HM Xu KX Yan C A chromosome-scale reference genome of the Banna miniature inbred pig Sci Data 2024 11 1345 10.1038/s41597-024-04201-3 39695204 PMC11655879

[17] Meng J Chen X Wang H Mi Y Zhou R Zhang H Porcine granulosa cell transcriptomic analyses reveal the differential regulation of lncRNAs and mRNAs in response to all-trans retinoic acid in vitro Anim Biosci 2025 38 267 77 10.5713/ab.24.0363 39210795 PMC11725750

[18] Chen KR Wang HY Kuo YC Lo YC Kuo PL A novel SEPT12 mutation, T96I, is associated with sperm head and annulus defects Front Cell Dev Biol 2025 12 1498013 10.3389/fcell.2024.1498013 39850804 PMC11756531

[19] Groenen MAM Archibald AL Uenishi H Analyses of pig genomes provide insight into porcine demography and evolution Nature 2012 491 393 8 10.1038/nature11622 23151582 PMC3566564

[20] Steels JD Estey MP Froese CD Reynaud D Pace-Asciak C Trimble WS Sept12 is a component of the mammalian sperm tail annulus Cell Motil Cytoskeleton 2007 64 794 807 10.1002/cm.20224 17685441

[21] van Son M Tremoen NH Gaustad AH Transcriptome profiling of porcine testis tissue reveals genes related to sperm hyperactive motility BMC Vet Res 2020 16 161 10.1186/s12917-020-02373-9 32456687 PMC7249385

[22] Ding X Yu W Liu M SEPT12 interacts with SEPT6 and this interaction alters the filament structure of SEPT6 in Hela cells BMB Rep 2007 40 973 8 10.5483/BMBRep.2007.40.6.973 18047794

[23] Lin YH Kuo YC Chiang HS Kuo PL The role of the septin family in spermiogenesis Spermatogenesis 2011 1 298 302 10.4161/spmg.1.4.18326 22332113 PMC3271641

[24] Kubota T Myung K Donaldson AD Is PCNA unloading the central function of the Elg1/ATAD5 replication factor C-like complex? Cell Cycle 2013 12 2570 9 10.4161/cc.25626 23907118 PMC3865047

[25] Park SH Kang N Song E ATAD5 promotes replication restart by regulating RAD51 and PCNA in response to replication stress Nat Commun 2019 10 5718 10.1038/s41467-019-13667-4 31844045 PMC6914801

[26] Castillo JP Rui H Basilio D Mechanism of potassium ion uptake by the Na+/K+-ATPase Nat Commun 2015 6 7622 10.1038/ncomms8622 26205423 PMC4515779

[27] Valdivia MM Hamdouch K Ortiz M Astola A CENPA a genomic marker for centromere activity and human diseases Curr Genom 2009 10 326 35 10.2174/138920209788920985 PMC2729997 20119530

[28] Okada M Cheeseman IM Hori T The CENP-H–I complex is required for the efficient incorporation of newly synthesized CENP-A into centromeres Nat Cell Biol 2006 8 446 57 10.1038/ncb1396 16622420

[29] Laity JH Lee BM Wright PE Zinc finger proteins: new insights into structural and functional diversity Curr Opin Struct Biol 2001 11 39 46 10.1016/S0959-440X(00)00167-6 11179890

[30] Henriques BJ Olsen RKJ Gomes CM Bross P Electron transfer flavoprotein and its role in mitochondrial energy metabolism in health and disease Gene 2021 776 145407 10.1016/j.gene.2021.145407 33450351 PMC7949704

[31] Greber BJ Ban N Structure and function of the mitochondrial ribosome Annu Rev Biochem 2016 85 103 32 10.1146/annurev-biochem-060815-014343 27023846

[32] Deng S Yang L Gao L Ning C Wang S Zhang W The effect of combined cryoprotectants on the cryotolerance of boar sperm Anim Biosci 2025 38 2111 24 10.5713/ab.24.0915 40302671 PMC12415376

[33] Urbańska K Orzechowski A Unappreciated role of LDHA and LDHB to control apoptosis and autophagy in tumor cells Int J Mol Sci 2019 20 2085 10.3390/ijms20092085 31035592 PMC6539221

[34] Maekawa M Ito C Toyama Y Stage-specific expression of mouse germ cell-less-1 (mGCL-1), and multiple deformations during mgcl-1 deficient spermatogenesis leading to reduced fertility Arch Histol Cytol 2004 67 335 47 10.1679/aohc.67.335 15700541

[35] Adam JC Pringle JR Peifer M Evidence for functional differentiation among Drosophila septins in cytokinesis and cellularization Mol Biol Cell 2000 11 3123 35 10.1091/mbc.11.9.3123 10982405 PMC14980

[36] Park C Choi Y Yoo S La H Hong K Analysis of DNA methylation changes following Cfp1 knockout in mouse spermatocytes Anim Biosci 2025 38 1570 9 10.5713/ab.24.0807 40045604 PMC12229914

[37] Mao Y Nickitenko A Duan X Crystal structure of the VHS and FYVE tandem domains of Hrs, a protein involved in membrane trafficking and signal transduction Cell 2000 100 447 56 10.1016/S0092-8674(00)80680-7 10693761

[38] Wang L Liu H Zhang X WFS1 functions in ER export of vesicular cargo proteins in pancreatic β-cells Nat Commun 2021 12 6996 10.1038/s41467-021-27344-y 34848728 PMC8632972

[39] Panfili E Mondanelli G Orabona C Novel mutations in the WFS1 gene are associated with Wolfram syndrome and systemic inflammation Hum Mol Genet 2021 30 265 76 10.1093/hmg/ddab040 33693650 PMC8091036

