# Supplementary material for: Functional characterization of porcine septin12 and its role in male reproduction
Source: Anim Biosci. 2026 Apr 2;39(7):250538. doi: 10.5713/ab.250538 (PMC13353119; doi:10.5713/ab.250538)
Supplement: Supplementary file 1 [file ab-250538-Supplementary-1.pdf]

Supplement 1. Sequence information of septin12s in *Artiodactyla* and other vertebrates.

Organism	Common name	Order	ID
Camelus dromedarius	Dromedary camel	*Artiodactyla*	XP_031303653.1
Camelus ferus	Wild camel		XP_014407281.2
Vicugna pacos	Alpaca		XP_006204397.1
Ovis aries	Sheep		XP_027817526.2
Capra hircus	Domestic goat		XP_017895586.1
Oryx dammah	Scimitar oryx		XP_040112124.1
Bos mutus	Wild yak		XP_005887877.1
Bubalus bubalis	Water buffalo		XP_006054964.1
Bos taurus	Cattle		NP_001091612.1
Bos indicus	Zebu		XP_019843631.1
Lagenorhynchus obliquidens	Pacific white-sided dolphin		XP_026973293.1
Globicephala melas	Long-finned pilot whale		XP_030739718.1
Tursiops truncatus	Bottlenose dolphin		XP_019784328.1
Orcinus orca	Killer whale		XP_004277309.1
Monodon monoceros	Narwhal		XP_029075565.1
Neophocaena asiaeorientalis asiaeorientalis	Yangtze finless porpoise		XP_024615209.1
Lipotes vexillifer	Baiji		XP_007453519.1
Balaenoptera musculus	Blue whale		XP_036703929.1
Sus scrofa	Wild boar		AFS88928.1
Equus caballus	Horse	*Perissodactyla*	XP_023472143.1
Equus asinus	Domestic ass		XP_014688714.2
Eumetopias jubatus	Steller sea lion	*Carnivora*	XP_027973219.1
Odobenus rosmarus divergens	Pacific walrus		XP_004403304.1
Halichoerus grypus	Grey seal		XP_035947337.1
Ailuropoda melanoleuca	Giant panda		XP_011229815.3
Canis lupus familiaris	Domestic dog		XP_022275807.1
Panthera tigris	Tiger		XP_042827514.1
Panthera leo	Lion		XP_042777824.1
Felis catus	Domestic cat		XP_006942423.2
Pan troglodytes	Chimpanzee	*Primates*	XP_001169473.2
Homo sapiens	Human		NP_653206.2
Pan paniscus	Bonobo		XP_054955498.1
Macaca fascicularis	Crab-eating macaque		XP_045237579.1
Macaca mulatta	Rhesus macaque		XP_002808333.2
Papio anubis	Olive baboon		XP_003916543.2
Chlorocebus sabaeus	Green monkey		XP_037845812.1
Macaca nemestrina	Pig-tailed macaque		XP_011716067.1
Rattus norvegicus	Rat	*Rodentia*	NP_001094335.1
Mesocricetus auratus	Golden hamster		XP_040604068.1
Mus musculus	Mouse		NP_001360874.1
