# Supplementary material for: Functional characterization of porcine septin12 and its role in male reproduction
Source: Anim Biosci. 2026 Apr 2;39(7):250538. doi: 10.5713/ab.250538 (PMC13353119; doi:10.5713/ab.250538)
Supplement: Supplementary file 2 [file ab-250538-Supplementary-2.pdf]

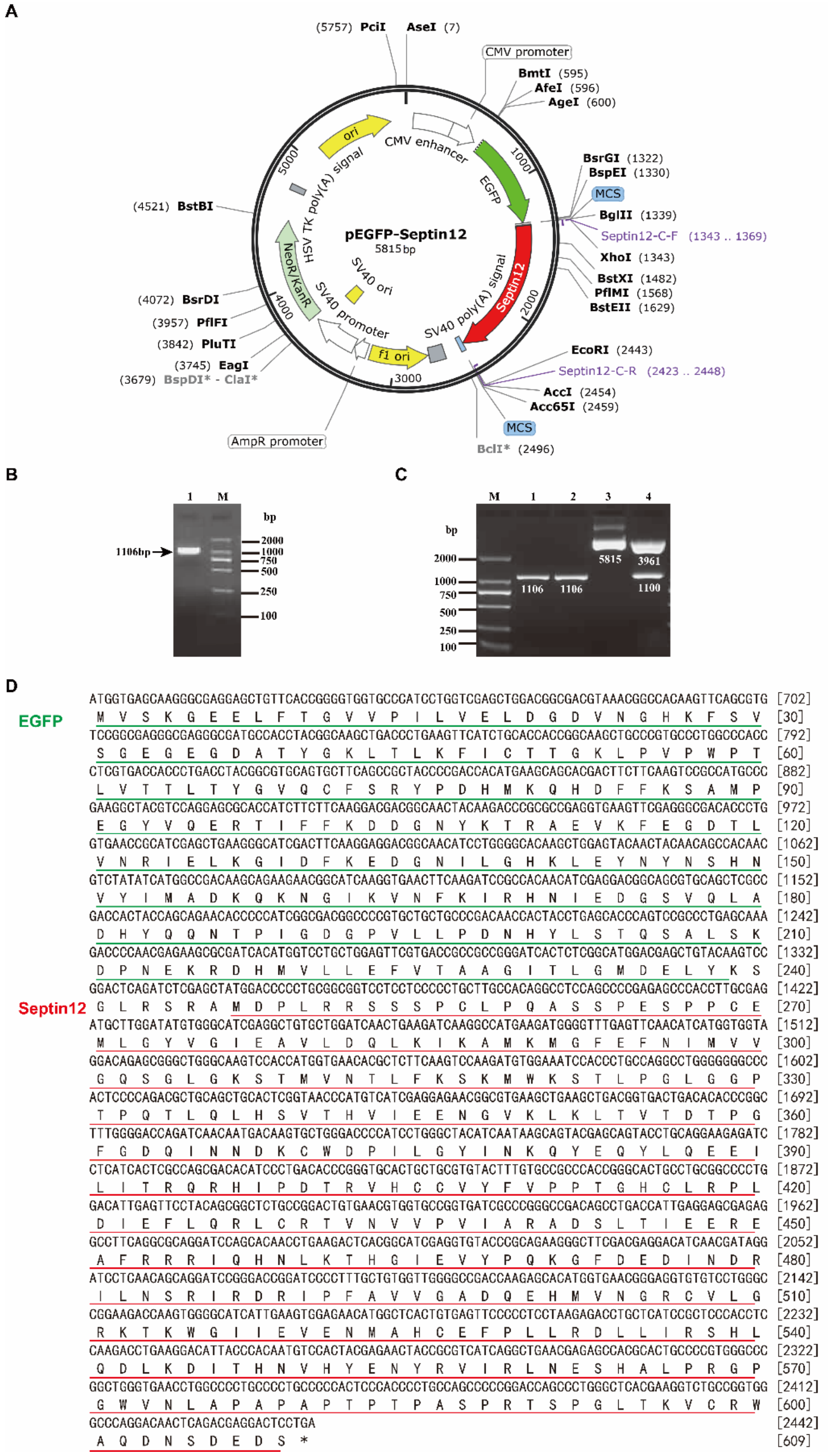

Supplement 2. Construction of co-expression vector pEGFP-septin12. (**A**) Schematic diagram of the expression vector construction. Green arrow and red arrow refer to the ORF of EGFP and septin12, respectively. (**B**) The PCR products of septin12 ORF were separated by electrophoresis on 1.5% agarose gel. (**C**) The constructed plasmids of pEGFP-septin12 were digested with *Xho* I and *Eco*R I and separated by electrophoresis on 1% agarose gel. (**D**) The ORF of the co-expression vector pEGFP-septin12.
