# Supplementary material for: Functional characterization of porcine septin12 and its role in male reproduction
Source: Anim Biosci. 2026 Apr 2;39(7):250538. doi: 10.5713/ab.250538 (PMC13353119; doi:10.5713/ab.250538)
Supplement: Supplementary file 3 [file ab-250538-Supplementary-3.pdf]

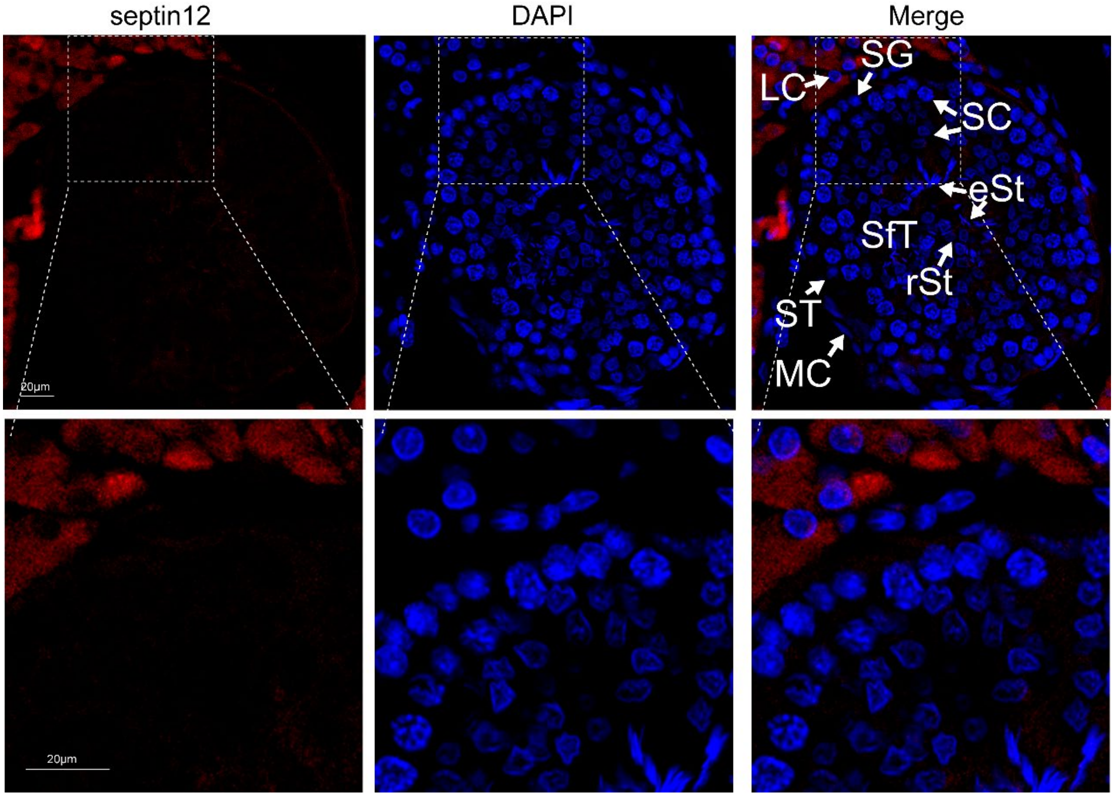

Supplement 3. Immunofluorescence staining of septin12 protein in pig testis sections. Nuclei were stained with DAPI (blue) and septin12 protein were shown in red. Arrowheads indicated the specific cell types identified based on their characteristic morphology and anatomical localization. SfT, seminiferous tubules; LC, Leydig cell; SG, spermatogonia; SC, spermatocyte; rST, round spermatid; eST, elongated spermatid; ST, Sertoli cell; MC, myoid cell. Leydig cells (LCs) were identified by their localization in the interstitial tissue surrounding the seminiferous tubules and by their round nuclei. Spermatocytes (SCs) were identified based on their intra-tubular localization above the basal spermatogonial layer and their distinct nuclear morphology, characterized by relatively large nuclei with condensed chromatin consistent with meiotic prophase. Elongated spermatids (eSTs) were distinguished by their luminal localization and their highly condensed, elongated nuclei. Septin12 immunoreactivity was predominantly detected in the interstitial (Leydig) cells, spermatocytes, and elongated spermatids.
