# Supplementary material for: Functional characterization of porcine septin12 and its role in male reproduction
Source: Anim Biosci. 2026 Apr 2;39(7):250538. doi: 10.5713/ab.250538 (PMC13353119; doi:10.5713/ab.250538)
Supplement: Supplementary file 4 [file ab-250538-Supplementary-4.pdf]

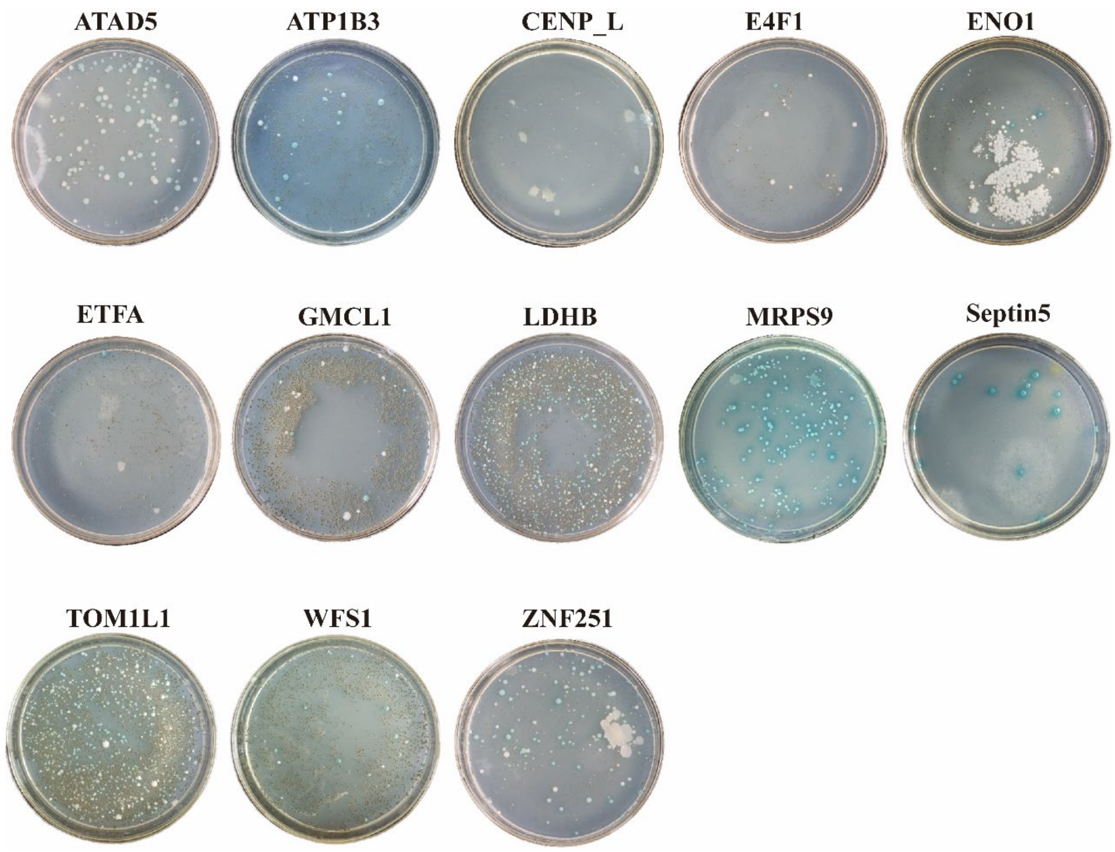

Supplement 4. Result of point-to-point verification of the 13 prey plasmids and the bait plasmid pGBKT7-septin12.
