# Supplementary material for: Functional characterization of porcine septin12 and its role in male reproduction
Source: Anim Biosci. 2026 Apr 2;39(7):250538. doi: 10.5713/ab.250538 (PMC13353119; doi:10.5713/ab.250538)
Supplement: Supplementary file 5 [file ab-250538-Supplementary-5.pdf]

Supplement 5. Gene ontology analysis on proteins interacting with septin12.

Gene	Entrze gene ID	Gene Name	Molecular function
ATAD5	100522895	ATPase family AAA domain containing 5	DNA binding, ATP binding, ATP hydrolysis activity, DNA clamp unloader activity
ATP1B3	100514493	ATPase Na+/K+ transporting subunit beta 3	ATPase activator activity
CENPL	100623697	centromere protein L	
E4F1	100517704	E4F transcription factor	RNA polymerase II cis-regulatory region sequence-specific DNA binding, DNA-binding transcription factor activity, RNA polymerase II-specific, DNA-binding transcription repressor activity, RNA polymerase II-specific, DNA-binding transcription activator activity, RNA polymerase II-specific, DNA-binding transcription factor activity, cAMP response element binding, metal ion binding, RNA polymerase II-specific DNA-binding transcription factor binding
ETFA	396615	electron transfer flavoprotein subunit alpha	electron transfer activity, oxidoreductase activity, flavin adenine dinucleotide binding
ENO1	100738870	enolase 1	magnesium ion binding, RNA polymerase II transcription regulatory region sequence-specific DNA binding, transcription corepressor binding, DNA-binding transcription repressor activity, RNA polymerase II-specific, transcription corepressor activity, phosphopyruvate hydratase activity, protein homodimerization activity, GTPase binding
GMCL1	100511872	germ cell-less 1, spermatogenesis associated	protein binding
LDHB	100621540	lactate dehydrogenase B	L-lactate dehydrogenase activity
MRPS9	100624945	mitochondrial ribosomal protein S9	RNA binding, structural constituent of ribosome
Septin5	100156312	septin 5	GTPase activity, GTP binding, identical protein binding, molecular adaptor activity,
TOM1L1	100522701	target of myb1 like 1 membrane trafficking protein	protein kinase binding, clathrin binding, protein kinase activator activity, phosphatidylinositol binding, ubiquitin binding
WFS1	100621070	wolframin ER transmembrane glycoprotein	protein binding, ubiquitin protein ligase binding, ATPase binding
ZNF251	100738561	zinc finger protein 251	RNA polymerase II cis-regulatory region sequence-specific DNA binding, DNA-binding transcription factor activity, RNA polymerase II-specific, DNA-binding transcription repressor activity, RNA polymerase II-specific, DNA binding, recombination hotspot binding, histone H3K4 methyltransferase activity, metal ion binding, histone H3K36 methyltransferase activity

Supplement 5. Gene ontology analysis on proteins interacting with septin12.

Gene	Biological process
ATAD5	Cell population proliferation, positive regulation of B cell proliferation, nuclear DNA replication, signal transduction in response to DNA damage, intrinsic apoptotic signaling pathway in response to DNA damage by p53 class mediator, isotype switching, positive regulation of DNA replication, positive regulation of isotype switching to IgG isotypes, regulation of mitotic cell cycle phase transition, negative regulation of intrinsic apoptotic signaling pathway in response to DNA damage by p53 class mediator, positive regulation of cell cycle G2/M phase transition
ATP1B3	intracellular sodium ion homeostasis, intracellular potassium ion homeostasis, sodium ion export across plasma membrane, potassium ion import across plasma membrane
CENPL	
E4F1	DNA replication, regulation of transcription by RNA polymerase II, regulation of mitotic cell cycle, embryonic, regulation of cell cycle process
ETFA	amino acid catabolic process, respiratory electron transport chain, fatty acid beta-oxidation using acyl-CoA dehydrogenase
ENO1	glycolytic process, response to virus, positive regulation of plasminogen activation, negative regulation of cell growth, positive regulation of muscle contraction, canonical glycolysis, negative regulation of hypoxia-induced intrinsic apoptotic signaling pathway, positive regulation of ATP biosynthetic process
GMCL1	regulation of DNA-templated transcription, germ cell development
LDHB	lactate metabolic process, pyruvate metabolic process, carboxylic acid metabolic process
MRPS9	translation
Septin5	regulation of exocytosis, cytoskeleton-dependent cytokinesis
TOM1L1	signal transduction, protein transport, positive regulation of protein autophosphorylation, negative regulation of mitotic nuclear division
WFS1	negative regulation of transcription by RNA polymerase II, kidney development, renal water homeostasis, visual perception, sensory perception of sound, endoplasmic reticulum unfolded protein response, positive regulation of protein ubiquitination, endoplasmic reticulum calcium ion homeostasis, ERAD pathway, glucose homeostasis, negative regulation of neuron apoptotic process, protein stabilization, positive regulation of calcium ion transport, calcium ion homeostasis, negative regulation of ATF6-mediated unfolded protein response, negative regulation of type B pancreatic cell apoptotic process
ZNF251	regulation of DNA-templated transcription, positive regulation of reciprocal meiotic recombination, hematopoietic stem cell homeostasis

Supplement 5. Gene ontology analysis on proteins interacting with septin12.

Gene	Cellular component
ATAD5	Nucleus, Elg1 RFC-like complex
ATP1B3	sodium:potassium-exchanging ATPase complex, basolateral plasma membrane, apical plasma membrane
CENPL	inner kinetochore, nucleus, chromosome
E4F1	Nucleus, spindle, nuclear body
ETFA	Mitochondrion, mitochondrial matrix, electron transfer flavoprotein complex
ENO1	phosphopyruvate hydratase complex, nuclear outer membrane, plasma membrane, cell cortex, cell surface
GMCL1	Nucleus, nuclear envelope, nuclear matrix
LDHB	Cytoplasm, mitochondrion, mitochondrial inner membrane
MRPS9	Nucleolus, mitochondrial small ribosomal subunit, ribosome
Septin5	plasma membrane, septin ring, synaptic vesicle, microtubule cytoskeleton, septin complex, cell division site
TOM1L1	Endosome, cytosol, membrane
WFS1	protein binding, ubiquitin protein ligase binding, ATPase binding
ZNF251	nucleus
